# Metaphors: the evolutionary journey from bidirectionality to unidirectionality

**DOI:** 10.1098/rstb.2020.0193

**Published:** 2021-05-10

**Authors:** David Gil, Yeshayahu Shen

**Affiliations:** ^1^ Max Planck Institute for the Science of Human History, Jena, Thüringen, Germany; ^2^ Department of Literature, Tel Aviv University, Tel Aviv, Israel

**Keywords:** metaphor, evolution, metaphor directionality, grammatical asymmetry, grammatical complexity, polity complexity

## Abstract

Metaphors, a ubiquitous feature of human language, reflect mappings from one conceptual domain onto another. Although founded on bidirectional relations of similarity, their linguistic expression is typically unidirectional, governed by conceptual hierarchies pertaining to abstractness, animacy and prototypicality. The unidirectional nature of metaphors is a product of various asymmetries characteristic of grammatical structure, in particular, those related to thematic role assignment. This paper argues that contemporary metaphor unidirectionality is the outcome of an evolutionary journey whose origin lies in an earlier bidirectionality. Invoking the Complexity Covariance Hypothesis governing the correlation of linguistic and socio-political complexity, the Evolutionary Inference Principle suggests that simpler linguistic structures are evolutionarily prior to more complex ones, and accordingly that bidirectional metaphors evolved at an earlier stage than unidirectional ones. This paper presents the results of an experiment comparing the degree of metaphor unidirectionality in two languages: Hebrew and Abui (spoken by some 16 000 people on the island of Alor in Indonesia). The results of the experiment show that metaphor unidirectionality is significantly higher in Hebrew than in Abui. Whereas Hebrew is a national language, Abui is a regional language of relatively low socio-political complexity. In accordance with the Evolutionary Inference Principle, the lower degree of metaphor unidirectionality of Abui may accordingly be reconstructed to an earlier stage in the evolution of language. The evolutionary journey from bidirectionality to unidirectionality in metaphors argued for here may be viewed as part of a larger package, whereby the development of grammatical complexity in various domains is driven by the incremental increases in socio-political complexity that characterize the course of human prehistory.

This article is part of the theme issue ‘Reconstructing prehistoric languages’.

## 1. Introduction

How did metaphors evolve? If figuring out the general ground plans of prehistorical languages may sometimes seem exceedingly challenging, tracing the evolution of specifically metaphorical constructions would appear to present an even more daunting task. Nevertheless, this paper suggests that it may indeed be possible to make inferences concerning the ways in which metaphors developed, within the more general course of language evolution.

Although metaphors are typically expressed through language, their basis is in conceptual structures, involving mappings between two distinct conceptual domains. As such, it makes sense to ask whether metaphorical thought is present among other animals, which lack language. At least one study suggests that it might be. In an investigation of rhesus monkeys, Merritt *et al*. [1] found that they share with humans a basic cross-domain mapping between space and time. In their experiments, Merritt *et al.* gave their participants a task that assessed the influence of an irrelevant dimension (time or space) on a relevant dimension (space or time) and found significant cross-domain interference effects, concluding that, in this particular domain at least, rhesus monkeys do indeed connect between space and time, thereby engaging in metaphorical thought.

However rudimentary they may be, the metaphorical abilities of rhesus monkeys thus highlight the potential interest in evolutionarily oriented investigations of metaphoricity. In particular, they raise the questions of how the metaphorical abilities of humans differ from those of other animals, and how they might have evolved to reach their current state. One perspective on these questions is that of Benítez-Burraco & Progovac [2], who argue that enhanced cross-domain mappings of the kind that underlie metaphorical abilities developed as a result of the process of self-domestication that took place in the course of human evolution. The present paper chooses to investigate the evolution of human metaphoricity through the prism of one of its most central properties, namely its *directionality*.

## 2. Metaphor directionality

Metaphor directionality lies at the heart of human metaphorical behaviour. In the case of the rhesus monkeys mentioned above, the observed effects were *bidirectional:* while in some conditions a temporal stimulus affected a spatial assessment, in other conditions a spatial stimulus influenced a temporal assessment. By contrast, among humans, metaphors are pervasively *unidirectional*. For example, while time is often conceptualized in terms of space, space is rarely or never conceptualized in terms of time; see Boroditsky [3], Casasanto & Boroditsky [4] and others. Thus, when the Merrit *et al*. [1] experiments were performed on humans, the spatial stimulus had a significantly greater impact on the temporal assessment than *vice versa*. In language, such space–time asymmetry is reflected by numerous expressions such as *long time*, and *on Monday*, in which a temporal expression (*time*, *Monday)*, is conceptualized by means of a spatial one (*long, on*), as contrasted with a dearth of mirror-image expressions in which a spatial expression would be conceptualized in terms of a temporal one.

Metaphor directionality is rooted in various conceptual hierarchies. For example, abstract entities tend to be described in terms of more concrete ones, the above-mentioned spatial/temporal asymmetries offering a particular case of this. Similarly, in accordance with other hierarchies, more animate entities tend to be described in terms of less animate ones and less prototypical entities in terms of more prototypical ones. A common way of describing metaphor unidirectionality is in terms of the twin notions of *target*, the concept being explicated, and *source*, the concept doing the explication. For example, in metaphors connecting space and time, the spatial expression is typically the source and the temporal expression its target. The unidirectionality of metaphors is a central theme in metaphor studies, as reflected in work by Lakoff & Johnson [5], Kogan *et al*. [6], Glucksberg & Keysar [7] and many others.

Notwithstanding the above, recent work suggests that the unidirectionality of metaphors rests on the foundations of a more basic bidirectionality. Logically speaking, metaphors appeal to cross-domain mappings that may potentially be defined in either direction, reflecting a fundamentally symmetric relation of similarity. Such symmetry is supported by a variety of psychophysical experiments revealing a clear bidirectional pattern for many hypothesized conceptual mappings; see Ĳzerman & Koole [8] for details. Moreover, in the online processing of metaphors, experiments by Wolff & Gentner [9] provide support for a two-stage model of metaphor comprehension, which is bidirectional for the first 500 ms before switching to unidirectional—leading them to posit a ‘double life' for metaphors, both bidirectional and unidirectional.

## 3. The role of grammar

A crucial factor underpinning metaphor unidirectionality is the presence of language, or, more specifically, grammar. As shown in Porat & Shen [10] and Shen & Porat [11], bidirectionality is manifest exclusively in non-verbal domains; as soon as grammar is introduced, unidirectionality comes to the fore. Following on this, in Gil & Shen [12] it is argued that metaphor unidirectionality is the outcome of thematic-role assignment and the asymmetric grammatical structures in which it is encoded. Specifically, in a variety of grammatical configurations, the metaphorical source assigns a thematic role to its target. For example, in an expression such as *long time*, the source expression *long* assigns the thematic role of theme to its target expression *time*.

The role of grammar in introducing metaphor directionality is manifest in two distinct ways, which we refer to here as *general* and *particular* directionality, respectively. General directionality represents the systematic way in which grammatical asymmetries predispose our cognitive processes towards correspondingly asymmetric conceptual hierarchies; it is, presumably, universal and invariant across different languages and cultures. By contrast, particular directionality effects are manifest in the ways in which the specific grammatical structures of languages provide further reinforcement to the same conceptual hierarchies; they may thus vary in their applicability both across different constructions within the same language, and across prima facie similar constructions in different languages.

The role of grammatical asymmetries in introducing metaphoricity and the distinction between general and particular directionality effects across different constructions within the same language, English, may be observed by comparing the following sentences involving a novel metaphor making use of the mapping between a spatial expression, *Nile*, and a temporal one, *Monday*:


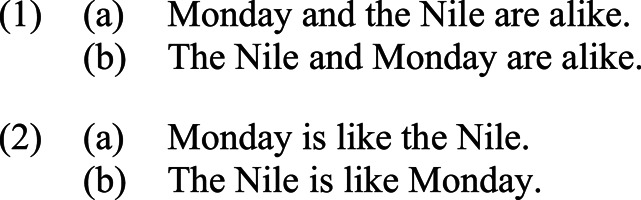


In accordance with general directionality, the mere presence of the grammatical medium activates the concrete/abstract conceptual hierarchy, yielding a preference for the more abstract *Monday* to be understood as explicated in terms of the more concrete *Nile*, appealing perhaps to the shared property of being long and slow-moving—under this interpretation, *Nile* is understood as the metaphorical source and *Monday* as its target. However, this preference comes up against the variegated grammatical constructions in (1) and (2), resulting in differential applications of particular directionality effects. In (1), the two terms occur in symmetric conjunctions, which, as shown by Fishman & Shen [13], are not metaphor-friendly constructions; as a result, the sentences do not provide for a natural expression of the desired metaphor. By contrast, in (2), the two terms occur in grammatically asymmetric constructions, with the first term in subject position, and the second term contained within the predicate. In general, subject–predicate constructions constitute one of the most common grammatical templates for the expression of metaphors; in such cases, the rules of English grammar dictate that the subject is interpreted as the target, while the term contained within the predicate is understood as its source. In (2a), these grammatical rules are congruent with the conceptual hierarchy, resulting in a well-formed metaphorical construction in which the two terms occur in the order that may accordingly be characterized as *canonical*. However, in (2b), the same grammatical rules conflict with the conceptual hierarchy, resulting in a non-canonically ordered metaphor that is more difficult to process and interpret. Thus, whereas in (2a), general and particular directionality effects reinforce one another, converging to produce a canonical metaphorical construction, in (2b) they diverge, resulting in a non-canonical metaphorical construction.

## 4. An evolutionary hypothesis

The role of grammar in introducing metaphor unidirectionality suggests that from an evolutionary perspective, bidirectionality is a living fossil, reflecting an earlier evolutionary stage, evinced by rhesus monkeys and other animals, in which grammar has not yet emerged. However, the presence of grammar also points towards a more fine-grained method of tracing the evolutionary path from bidirectionality to unidirectionality, appealing to patterns of cross-linguistic variation. In particular, given the role of grammar in inducing particular directionality effects, as in (1) and (2) above, it stands to reason that languages with different grammars may also perhaps be associated with different degrees of metaphor directionality. Moreover, if and when such differences are found, they may potentially provide a model for the evolution of metaphor directionality.

In Gil [14], a method is proposed for making use of contemporary cross-linguistic variation in order to reconstruct earlier stages in the evolution of language. The method is based on the observation that in several distinct domains, linguistic complexity covaries with cultural and socio-political complexity: the *Complexity Covariance Hypothesis*. In such cases, it may be inferred that the simpler linguistic structures are evolutionarily prior to their more complex counterparts: the *Evolutionary Inference Principle*. This paper applies this method in order to adduce empirical support for the reconstruction of metaphor bidirectionality to an earlier stage in the evolution of human language.

The first step in the argumentation rests on the observation that metaphor bidirectionality is less complex than metaphor unidirectionality. This is because the relationship between the two is privative. Whereas bidirectionality may be represented as a simple symmetric juxtaposition of two terms, X ∼ Y, unidirectionality adds an additional element to the mix, namely the characterization of one of the two terms as target and the other as source; see Gil & Shen [12] for further discussion.

In order to invoke the Evolutionary Inference Principle, the greater complexity of metaphor unidirectionality must be shown to correlate with some extra-linguistic measure of complexity. Whereas Gil [14] makes use of language family size as a measure of cultural and socio-political complexity, the present paper makes reference to the following scale of Polity Complexity, first introduced in Gil & Shen [12]:


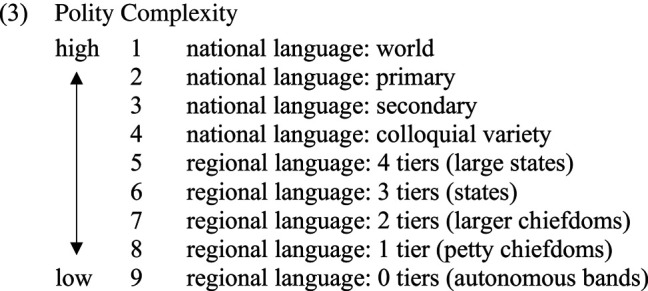


The above scale is of a hybrid nature, combining several measures of polity complexity. First is a basic dichotomy between national and regional languages. National languages are further distinguished with respect to more specific features pertaining to the language's functions and status. And regional languages are classified in terms of the complexity of their associated societies as reflected in the number of levels of ‘jurisdictional hierarchy beyond local community', as defined in the D-Place database [15].

With this in hand, we may now formulate an empirical hypothesis relating to metaphor directionality and polity complexity:


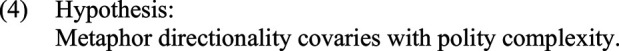


The above hypothesis predicts that languages with greater metaphor unidirectionality will be associated with polities of greater complexity in accordance with the scale in (3). If the hypothesis in (4) turns out to be supported, then this would provide further support for the reconstruction of metaphor bidirectionality to an earlier stage in the evolution of human language. In the next section, we provide some preliminary empirical support for the above hypothesis.

## 5. The Context Experiment

In order to test the hypothesis in (4), we adapted the Context Experiment first developed in Porat & Shen [10]. In the Context Experiment, participants are presented with 16 stimuli such as the following:


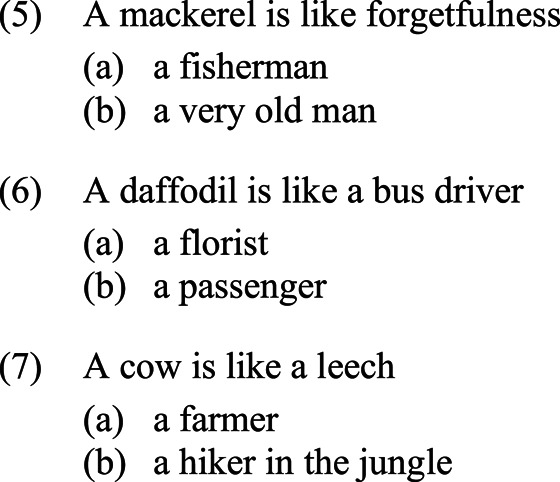


Each stimulus consists of a metaphor asserting the similarity of two generically referring terms, followed by two expressions referring to people. The participants' task is to choose which of the two people is more likely to utter the metaphor.

The metaphors are all novel and are not necessarily supposed to ‘make sense'. The choice of novel rather than conventionalized metaphors is intended to control for possible effects stemming from participants' differential levels of education and familiarity with the metaphors. The metaphors are all presented in the non-canonical order, that is to say, in violation of metaphor unidirectionality and the underlying conceptual hierarchies. For example, in (5), it would be more natural to say that *Forgetfulness is like a mackerel*, since concrete objects are more likely to explicate abstract ones than *vice versa*. In the experiment, six stimuli make reference to the concrete/abstract hierarchy as in (5), six to the animacy hierarchy as in (6) and four to the prototypicality hierarchy as in (7). Of the two potential speakers that the participants must choose from, one is associated with the first of the two terms while the other is associated with the second term. (While as presented in (5)–(7) above, the (a) speaker is associated with the first term and the (b) speaker with the second term, in the actual experiment the relative order of the two speakers is randomized.)

The Context Experiment pits grammatical structures and particular directionality effects against conceptual hierarchies and the general directionality effect, posing participants with a dilemma. For example, in (5), the grammatical structure points towards the fisherman as being the speaker, since he is associated with the subject of the sentence, referring to a mackerel; however, the concrete/abstract hierarchy suggests instead that the very old man should be the speaker, since he is connected with the abstract concept of forgetfulness. Thus, by examining participants' responses to choices such as this, we may assess the contribution of grammatical asymmetries to metaphor directionality.

The Context Experiment provides a tool for testing the covariation of metaphor directionality and polity complexity in accordance with the hypothesis in (4). Given the universality of the general directionality effect, the experiment provides a measure of the force of particular directionality effects as an additional contribution to overall metaphor directionality, pointing towards the following prediction:


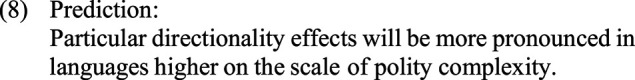


In the discussion of examples (5)–(7) above, a substantial particular directionality effect was presupposed for English; such an effect is to be expected given the status of English as a world language and its position occupying rank 1 at the top of the polity complexity scale in (3). However, in order to test the prediction in (8), particular directionality effects must be tested in a substantial sample of languages.

The experimental programme is currently in progress. At the time of writing, we have run the experiment in 10 different languages associated with varying levels of polity complexity, and initial results provide substantial support for the prediction in (8). For reasons of time and space, this paper presents results from just two of the languages, *Hebrew* and *Abui*. Hebrew, with several million native speakers, is the national language of Israel, and accordingly occupies rank 2 on the polity complexity scale, as a primary national language. Abui, described by Kratochvíl [16] and Saad [17], belongs to the Timor–Alor–Pantar language family, and is spoken by some 16 000 people on the Indonesian island of Alor; it occupies rank 8 on the polity complexity scale, as a regional language with just one tier of jurisdictional hierarchy, petty chiefdoms. Thus, Hebrew and Abui offer a clear-cut contrast between languages of high and low polity complexity.

Some example stimuli, the counterparts of those in (5)–(7) above, are presented below, for Hebrew in (9)–(11), and for Abui in (12)–(14):


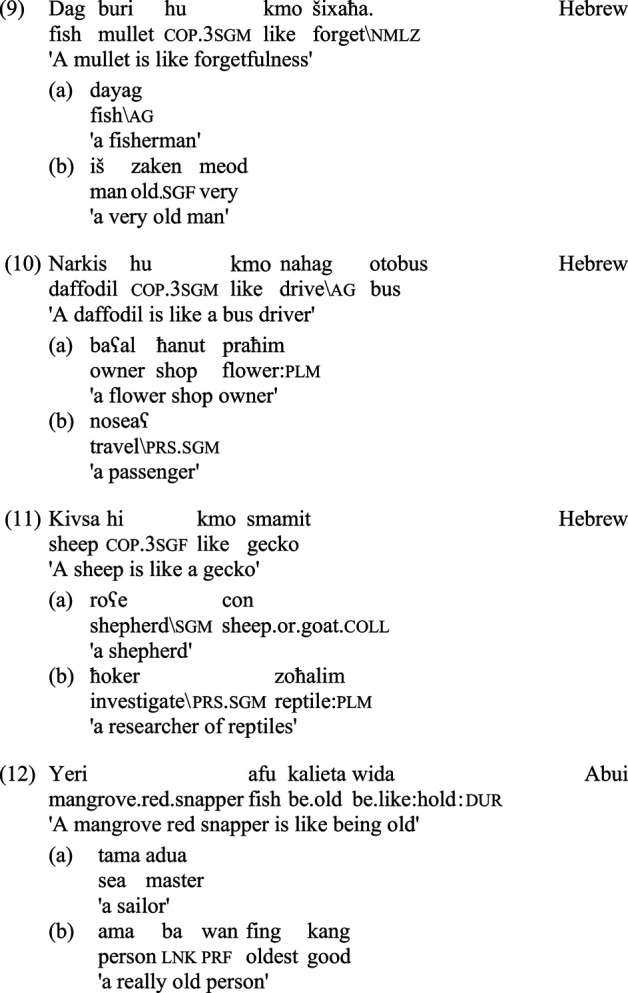



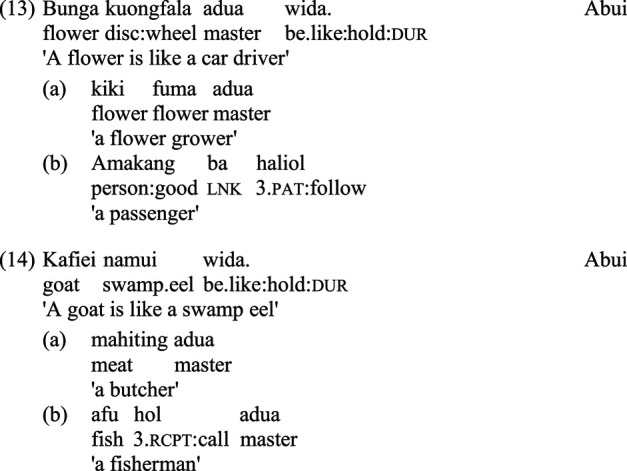


For each of the two languages, the metaphors are couched in the most natural construction available for the asymmetric expression of similarity: while for Hebrew this involves a copula followed by the word *kmo* ‘like', for Abui this consists of the complex verbal expression *wida*. As evident in the above examples, certain adjustments had to be made in order to construct comparable stimuli in languages associated with very different cultures and ecologies. To cite just one example, stimuli (7) in English, (11) in Hebrew and (14) in Abui are all intended to contrast a prototypical animal with a non-prototypical one; however, the kinds of animals and their relative degrees of prototypicality differ from place to place, as do the potentially available speakers who might be associated, through diverse cultural conventions, with the animals in question.

In addition to the 16 experimental stimuli, six additional distractor stimuli were included in the experiment. These distractors contained a metaphor in canonical order, followed by two potential speakers: while one of the speakers was associated with the first term, the one in subject position functioning as the target of the metaphor, the second speaker was not associated with either of the two terms. Competent participants were therefore expected to choose the speaker associated with the first term and not the speaker associated with neither of the terms; participants failing to do so consistently were excluded as not having properly understood or sufficiently carefully performed the experimental task. The experiment was conducted online, using Google Forms, with the link being distributed mostly via Facebook and Twitter.

The results of the experiment in Hebrew and Abui are summarized in table 1. In table 1, the first column specifies the language, the second column the total number of participants, and the third column the number of participants who passed all six distractors. The fourth and final column presents the responses of the participants who passed all six distractors: summing over all 16 experimental stimuli, it shows the percentage of grammatically canonical responses in which participants chose the speaker associated with the subject target expression, for example, the (a) choice in (9)–(14). These percentages thus represent the responses in which a particular directionality effect won out over the general directionality effect.

**Table 1.** Context experiment results.

language	participants: total	participants: passed all distractors	% of grammatically canonical responses
Hebrew	97	67	81.0
Abui	59	15	47.1

As evident in table 1, the particular directionality effect is much stronger in Hebrew, at 81.0%, than it is in Abui, at 47.1%; this difference is statistically significant at *p* < 0.00001 (according to a one-tailed *t*-test). Whereas in Hebrew grammatical structure trumps conceptual hierarchies, in Abui the two are more evenly matched. (Note that the restriction of the analysis in table 1 to participants who passed all six distractors rules out an alternative hypothesis to the effect that the near 50–50 score of the Abui participants might have been due to their guessing randomly, owing to their lack of proper understanding of the experimental task.)

Given the relative positions of Hebrew and Abui on the polity complexity scale, the results bear out the prediction in (8), namely that particular directionality effects will be more pronounced in languages higher on the scale of polity complexity. In turn, these results lend initial support to the hypothesis in (4), to the effect that metaphor directionality covaries with polity complexity. Invoking the Evolutionary Inference Principle, the results of the Context Experiment thus provide further support for the reconstruction of metaphor bidirectionality to an earlier stage in the evolution of human language.

## 6. Grammaticalization of thematic roles

As is evident in (9)–(14), the experimental stimuli in Hebrew and Abui differ from each other not only with respect to the words that they use, but also with regard to the grammatical structures in which these words occur. One obvious difference is in word order: while in Hebrew, with basic subject–verb–object word order, the expression *hu/hi kmo* ‘is like' occurs between the two terms being compared, in Abui, with basic subject–object–verb word order, the expression *wida* ‘is like' occurs at the end of the clause, following the two terms being compared. A second difference pertains to flagging: whereas in Hebrew the second term in the comparison is marked with *kmo* ‘like', in Abui both terms occur in bare form, without any adposition or case-marking.

**Figure 1.**
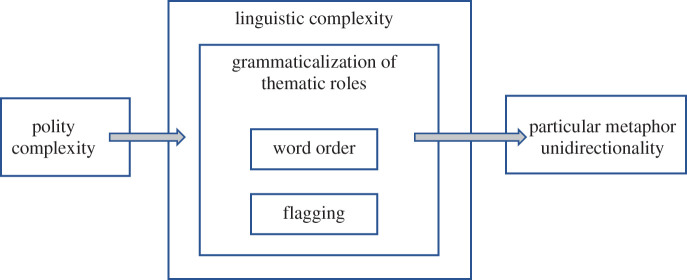
Polity complexity, grammar and particular metaphor unidirectionality.

The covariance between polity complexity and particular metaphor unidirectionality, as evidenced by the results of the Context Experiment, is not direct, but rather mediated by grammatical properties of the respective languages such as the ones mentioned above. A schematic representation of the relationship between polity complexity, grammar, and particular metaphor unidirectionality is provided in figure 1.

In figure 1, the first of the two arrows represents the Complexity Covariance Hypothesis, whereby cultural and socio-political complexity correlate with linguistic complexity in a variety of domains. One of these domains is the degree of grammaticalization of thematic roles, with a higher degree of grammaticalization instantiating greater linguistic complexity. The grammaticalization of thematic roles is reflected, in turn, by a variety of morphosyntactic features, foremost among which are word order and flagging, where more rigid word order and more flagging represent a greater degree of grammaticalization of thematic roles. As shown by the second arrow in figure 1, it is the degree of grammaticalization of thematic roles that governs particular metaphor directionality, as measured by the Context Experiment.

The degree of grammaticalization of thematic roles is an abstract property of grammars that is manifest in a variety of ways, of which particular metaphor unidirectionality is just one. A more direct measure of the degree of grammaticalization of thematic roles is provided by the ongoing Association Experiment, some preliminary results of which have been published in Gil [18,19] and Gil & Shen [12] (see also brief mention in the concluding section of Gil [14]). The Association Experiment measures the degree to which the assignment of thematic roles such as agent, patient and so forth is grammaticalized in specific constructions making use of morphosyntactic devices such as word order and flagging. For example, in one of the stimuli, participants are presented with a picture of a woman pushing a car, together with a sentence corresponding to *The car is pushing the woman*, and are asked to judge whether the sentence constitutes an appropriate description of the picture. English-speaking participants will say ‘no', because the sentence is ‘round the wrong way', English conveying the distinction between agent and patient thematic roles by means of word order. But what of the corresponding sentences in Hebrew and Abui? In Hebrew, speakers will also reject the sentence, *Hamexonit doħefet et hai*š*a*, albeit for a different reason, namely the presence of the accusative preposition *et* flagging *hai*š*a* ‘the woman', marking it as bearing the role of patient, and thereby conflicting with the picture. By contrast, in Abui, most speakers will accept the corresponding sentence, *Oto mayool hasuonra*, as being a true description of the picture. Summing over the experimental results for this and all the other stimuli of the Association Experiment, while Hebrew speakers exhibit a 6.7% rate of acceptability of such alternative interpretations, reflecting a high degree of grammaticalization of thematic-role assignment, speakers of Abui exhibit a much greater readiness, at 71.8%, to accept interpretations involving alternative assignments of thematic roles, suggesting that the degree of grammaticalization of thematic-role assignment in Abui is substantially lower. Thus, the results of the Association Experiment in Hebrew and Abui dovetail nicely with those of the Context Experiment, both reflecting the greater degree of grammaticalization of thematic roles in Hebrew in comparison with Abui. Moreover, just like the Context Experiment, the Association Experiment reveals a clear and significant correlation with polity complexity, as per the scale in (3) above: in a sample of 69 different languages, including Hebrew and Abui, languages whose polities are of greater complexity exhibit a greater degree of grammaticalization of thematic-role assignment, again in accordance with the Complexity Covariance Hypothesis.

Thus, appealing to the Evolutionary Inference Principle, the developmental journey from bidirectionality to unidirectionality in metaphors, argued for in this paper, may be viewed as part of a larger package, whereby the evolution of grammatical complexity and the grammaticalization of thematic roles, reflected also by particular metaphor directionality, are driven by the incremental increases in polity complexity that characterize the course of human prehistory.

## 7. Conclusion

Although of a preliminary nature, involving just two languages, the results of the Context Experiment in Hebrew and Abui provide strong initial support for the *Complexity Covariance Hypothesis,* the positive correlation between complexity in the linguistic and the cultural and socio-political domains. Invoking the Evolutionary Inference Principle, such covariance points towards the reconstruction of an earlier metaphor bidirectionality, followed by an evolutionary journey from bidirectionality to unidirectionality.

**Figure 2.**
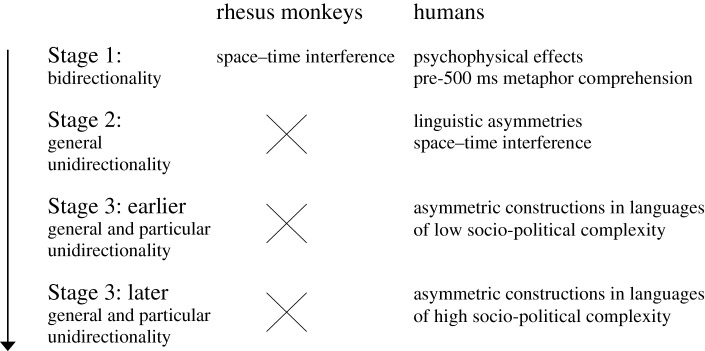
The evolution of metaphor unidirectionality.

In conjunction with previous studies mentioned earlier, the results of the Context Experiment support the existence of three stages in the evolutionary journey from bidirectionality to unidirectionality, with the third stage being further differentiated into earlier and later substages. These stages are represented in figure 2.

Stage 1, pure bidirectionality, is pre-linguistic: it is represented in rhesus monkeys by the space–time interference found by Merritt *et al*. [1], and in humans by the psychophysical experiments summarized by Ĳzerman & Koole [8] and the first pre-500 ms stage of metaphor comprehension found by Wolff & Gentner [9]. Stage 2, general unidirectionality, results from the emergence of asymmetric grammar as a universal, specifically human ability: its effect is manifest not only in the numerous studies of linguistically expressed metaphor directionality by Lakoff & Johnson [5], Kogan *et al*. [6], Glucksberg & Keysar [7] and others, but also in the pre-linguistic space–time interference found in humans by Merritt *et al*. [1]. Stage 3, general and particular unidirectionality, is the product of linguistic diversification: while all languages presumably exhibit some degree of particular directionality, the Context Experiment shows that such effects are stronger in Hebrew, a language of high socio-political complexity, than in Abui, a language of low socio-political complexity, thereby providing preliminary support for the conclusion that, within Stage 3, languages of higher socio-political complexity occupy a later substage than languages of lower socio-political complexity.

Thus, figure 2 provides a representation of the multiple stages in the evolutionary journey of metaphors from bidirectional to unidirectional. While the metaphorical abilities of humans are obviously more advanced than those of rhesus monkeys, the degree of metaphorical directionality in humans varies in accordance with the task involved and also the socio-political complexity of the language, with earlier evolutionary stages of directionality still present as living fossils in contemporary human metaphorical behaviour.

## Data accessibility

The data will be linked to a repository currently under construction. In the meantime, to review the data, please contact the first author.

## References

[1] Merritt DJ, Casasanto D, Brannon EM. 2010 Do monkeys think in metaphors? Representations of space and time in monkeys and humans. Cognition **117**, 191-202. (10.1016/j.cognition.2010.08.011) 20846645 PMC2952654

[2] Benítez-Burraco A, Progovac L. 2021 Language evolution: examining the link between cross-modality and aggression through the lens of disorders. Phil. Trans. R. Soc. B **376**, 20200188. (10.1098/rstb.2020.0188) 33745319 PMC8059641

[3] Boroditsky L. 2000 Metaphoric structuring: understanding time through spatial metaphors. Cognition **75**, 1-28. (10.1016/S0010-0277(99)00073-6) 10815775

[4] Casasanto D, Boroditsky L. 2008 Time in the mind: using space to think about time. Cognition **106**, 579-593. (10.1016/j.cognition.2007.03.004) 17509553

[5] Lakoff G, Johnson M. 1980 Metaphors we live by. Chicago, IL: University of Chicago Press.

[6] Kogan N, Chadrow M, Harbour H. 1989 Developmental trends in metaphoric asymmetry. Metaphor Symb. Act. **4**, 71-91. (10.1207/s15327868ms0402_1)

[7] Glucksberg S, Keysar B. 1990 Understanding metaphorical comparisons: beyond similarity. Psychol. Rev. **97**, 3-18. (10.1037/0033-295X.97.1.3)

[8] Ĳzerman H, Koole SL. 2011 From perceptual rags to metaphoric riches—bodily, social, and cultural constraints on sociocognitive metaphors: comment on Landau, Meier, and Keefer (2010). Psychol. Bull. **137**, 355-361. (10.1037/a0022373) 21355634

[9] Wolff P, Gentner D. 2011 Structure-mapping in metaphor comprehension. Cogn. Sci. **35**, 1456-1488. (10.1111/j.1551-6709.2011.01194.x) 21929665

[10] Porat R, Shen Y. 2017 The journey from bidirectionality to unidirectionality. Poetics Today **38**, 123-140. (10.1215/03335372-3716252)

[11] Shen Y, Porat R. 2017 Metaphorical directionality: the role of language. In Metaphor: embodied cognition and discourse (ed. B Hampe), pp. 62-81. Cambridge, UK: Cambridge University Press.

[12] Gil D, Shen Y. 2019 How grammar introduces asymmetry into cognitive structures: compositional semantics, metaphors and schematological hybrids. Front. Psychol. **10**, 1664-1678. (10.3389/fpsyg.2019.02275) 31681084 PMC6812659

[13] Fishman A, Shen Y. In preparation The (bi-directional) relation between grammatical asymmetry and metaphoricity.

[14] Gil D. 2021 Tense-aspect-mood marking, language-family size and the evolution of predication. Phil. Trans. R. Soc. B **376**, 20200194. (10.1098/rstb.2020.0194) 33745313 PMC8059509

[15] Kirby KR et al. 2016 D-PLACE: a global database of cultural, linguistic and environmental diversity. PLoS ONE **11**, e0158391. (10.1371/journal.pone.0158391) 27391016 PMC4938595

[16] Kratochvíl F. 2007 A grammar of Abui: a Papuan language of Alor. Utrecht, The Netherlands: LOT.

[17] Saad G. 2020 Variation and change in Abui, the impact of Alor Malay on an indigenous language of Indonesia. Utrecht, The Netherlands: LOT.

[18] Gil D. 2007 Creoles, complexity and associational semantics. In Deconstructing Creole: new horizons in language creation (eds U Ansaldo, SJ Matthews), pp. 67-108. Amsterdam, The Netherlands: John Benjamins.

[19] Gil D. 2008 How complex are isolating languages? In Language complexity: typology, contact, change (eds F Karlsson, M Miestamo, K Sinnemäki), pp. 109-131. Amsterdam, The Netherlands: John Benjamins.

